# Environmental exposures associated with elevated risk for autism spectrum disorder may augment the burden of deleterious de novo mutations among probands

**DOI:** 10.1038/s41380-021-01142-w

**Published:** 2021-05-17

**Authors:** Kealan Pugsley, Stephen W. Scherer, Mark A. Bellgrove, Ziarih Hawi

**Affiliations:** 1 Turner Institute for Brain and Mental Health, School of Psychological Sciences, Monash University, Melbourne, VIC Australia; 2 The Centre for Applied Genomics and Genetics and Genome Biology, The Hospital for Sick Children, Toronto, ON Canada; 3 McLaughlin Centre and Department of Molecular Genetics, University of Toronto, Toronto, ON Canada

**Keywords:** Genetics, Psychology, Molecular biology

## Abstract

Although the full aetiology of autism spectrum disorder (ASD) is unknown, familial and twin studies demonstrate high heritability of 60–90%, indicating a predominant role of genetics in the development of the disorder. The genetic architecture of ASD consists of a complex array of rare and common variants of all classes of genetic variation usually acting additively to augment individual risk. The relative contribution of heredity in ASD persists despite selective pressures against the classic autistic phenotype; a phenomenon thought to be explained, in part, by the incidence of spontaneous (or de novo) mutations. Notably, environmental exposures attributed as salient risk factors for ASD may play a causal role in the emergence of deleterious de novo variations, with several ASD-associated agents having significant mutagenic potential. To explore this hypothesis, this review article assesses published epidemiological data with evidence derived from assays of mutagenicity, both in vivo and in vitro, to determine the likely role such agents may play in augmenting the genetic liability in ASD. Broadly, these exposures were observed to elicit genomic alterations through one or a combination of: (1) direct interaction with genetic material; (2) impaired DNA repair; or (3) oxidative DNA damage. However, the direct contribution of these factors to the ASD phenotype cannot be determined without further analysis. The development of comprehensive prospective birth cohorts in combination with genome sequencing is essential to forming a causal, mechanistic account of de novo mutations in ASD that links exposure, genotypic alterations, and phenotypic consequences.

## Introduction

Autism spectrum disorder (ASD) is a pervasive neurodevelopmental condition estimated to affect ~1–1.5% of the global population [1, 2]. The behavioural phenotype of the disorder is characterised by early-onset dysfunction in social-communicative reciprocity and behavioural inflexibility [3], resulting in clinically significant impairment across a range of interpersonal, academic, and occupational contexts. Although age-related gains in adaptive functioning may attenuate symptomology in a subset of cases over time [4], the core deficits associated with childhood ASD minimally remit across the lifespan [5].

**Fig. 1:**
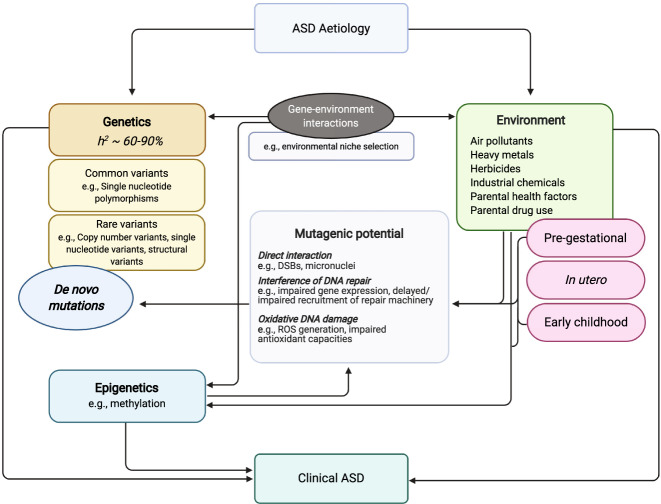
Diagrammatic representation of the interplay between genetic and environmental risk factors in the aetiology of ASD. Both heritable and non-heritable factors can independently and reciprocally influence the development of ASD symptomatology. Up to 5–15% of ASD probands possess risk-associated de novo mutations, indicating the significance of non-familial genetic variability in determining disorder risk. The mutagenic/genotoxic potential of non-heritable factors associated with ASD suggests that these toxicants may play a role in the elicitation of spontaneous mutations. Figure created with BioRender.com.

A strong contribution of heritable factors in the aetiology of ASD is supported by disproportionately increased risk of onset among first-degree relatives of probands [6, 7] and monozygotic twin concordance exceeding 60–90% [8, 9]. The genetic architecture of the disorder consists of a complex array of both rare (e.g., copy-number and single nucleotide variants, chromosomal abnormalities) [10] and common single nucleotide polymorphisms [2] acting additively to augment individual ASD risk (Fig. 1). The relative contribution of these mutations to the aetiology of the disorder is estimated at 2.5–15% and 12–52% [11, 12], respectively, with more recent evidence supporting the role of tandem repeat variations as additional, and incredibly salient, components of the ASD genotype (see section *Elevated genomic sensitivity to exposure-induced mutagenesis*). In several cases of syndromic ASD, a single genetic mutation seems sufficient to induce symptom onset [13], indicating that the disorder phenotype coincides disruption in important loss-of-function intolerant genes.

The contribution of heredity in ASD persists despite strong selective pressures against the deleterious genetic events associated with disorder onset. Interestingly, the reproductive challenges faced by individuals with ASD are not physical, but instead mostly social [14, 15]. Fecundity is consequently reduced among affected individuals [16], limiting the potential transmission of putative risk variants to offspring in subsequent generations. Despite this, the prevalence of ASD has demonstrated stability or increases over time, with non-heritable aetiological factors insufficiently compensating for loss of high-risk genetic variants from the reproductive gene pool [17, 18].

Preservation of the genetic liability of ASD despite reduced transmission of risk variants has been theorised to occur, in part, due to spontaneous de novo mutations [12, 19] (see Table 1). Depending on the study, which includes the complexity of the ASD phenotype, it is estimated that between 5 and 15% of ASD probands carry de novo mutations presumed to be involved in the disease [12], with a greater burden of de novo risk observed in simplex families without prior history of neurodevelopmental concerns [20]. Spontaneous genetic events are therefore more likely to represent important contributory factors to sporadic cases of ASD. Notably, although the rate of genomic de novo mutations is approximately equal between affected and unaffected familial trios, transmission of pathogenic mutations in important loss-of-function intolerant genes and gene pathways is observably higher among ASD probands [21–23], thereby consituting a critical feature of disorder aetiology.

**Table 1:** Key supporting evidence for the contribution of de novo mutations to the genetic aetiology of ASD.

Study	Sample description^a^	Sequencing methodology	Summary of relevant key findings
Sebat et al. (2007) [23]	264 families (118 simplex, 47 multiplex, 99 control)	Comparative genomic hybridization	The frequency of de novo CNVs was significantly higher in ASD-affected relative to unaffected children, and nominally higher in simplex relative to multiplex families
Marshall et al. (2008) [22]	427 ASD families (237 simplex, 189 multiplex)	Microarray analysis & karyotyping	De novo CNVs were more frequently detected among ASD cases from simplex families compared to those from multiplex families, however both exceeded the de novo rate expected in control families. Karyotyping revealed a small number of additional balanced cytogenic anomalies of de novo origin among ASD probands (simplex and multiplex)
Pinto et al. (2010) [118]	996 ASD probands, 1287 matched controls	Genome-wide microarray analysis	There was a significant 1.19-fold increase in the burden of rare genic CNVs, including de novo variants, for ASD cases relative to controls. Over 5% of ASD cases possessed at least one de novo CNV, with >0.6% carrying two or more de novo variants
Levy et al. (2011) [119]	915 families (889 ASD probands, 895 control siblings)	Comparative genomic hybridization	De novo CNVs were detected in 7.9% of ASD probands versus 2.0% of all siblings, with CNVs occurring significantly more frequently in genic regions in ASD-affected compared to unaffected siblings
O’Roak et al. (2011) [120]	20 ASD trios	Exome sequencing	The observed rate of de novo protein coding mutations was slightly higher among ASD probands than expected in the general population. Potentially causative de novo mutations were detected in four of the most severely affected cases according to IQ and calibrated severity scores
Sanders et al. (2011) [121]	1124 families (872 quads, 8252 trios^b^)	Genome-wide microarray analysis	De novo CNVs were significantly more frequent in ASD probands compared to unaffected siblings (OR = 3.5), with CNVs being larger on average and harbouring a larger number of genes
Iossifov et al. (2012) [122]	343 ASD quads^b^	Exome sequencing	Likely gene-disrupting de novo mutations, including nonsense, splice site, and frameshift mutations were twice as frequent among ASD probands relative to control siblings
Sanders et al. (2012) [19]	225 families (200 quads, 25 trios^b^)	Exome sequencing	The frequency of nonsynonymous de novo SNVs, including nonsense SNVs and splice site altering SNVs, were higher in affected relative to unaffected siblings, with a significant increase in the proportion of gene-disrupting compared to silent mutations among probands versus siblings (OR = 1.93)
Jiang et al. (2013) [123]	32 trios^b^	Genome sequencing	De novo SNVs likely to contribute to ASD presentation were detected in 19% of examined probands, affecting both known and novel ASD risk genes
De Rubeis et al. (2014) [55]	2270 ASD trios^b^, 510 control trios	Exome sequencing	De novo loss-of-function mutations were observed significantly more frequently in ASD trios relative to matched control trios
Dong et al. (2014) [124]	787 families (602 quads, 185 trios^b^)	Exome sequencing	Spontaneous de novo frameshift indels were found to contribute to ASD risk among probands (OR = 1.6)
Iossifov et al. (2014) [12]	2517 families (2508 ASD probands, 1911 control siblings)	Exome sequencing	The ascertainment differentials for the rates of likely gene-disrupting and missense de novo mutations were significant when comparing ASD probands to unaffected siblings, contributing to 12% and 9% of ASD diagnoses, respectively
O’Roak et al. (2014) [125]	3486 ASD probands, 2493 control siblings	Exome sequencing	Observed an elevated rate of de novo mutations in candidate ASD genes among affected relative to unaffected siblings, with a 2.7-fold greater rate of protein-altering de novo mutations among ASD probands
Pinto et al. (2014) [21]	2446 ASD families (simplex & multiplex), 4768 control subjects	Genome-wide microarray analysis	Reported a significant 1.41-fold excess of genic de novo CNVs of greater average length among ASD cases compared to controls. ASD probands were more likely to possess de novo variation in ASD candidate genes and gene networks related to processes of neurodevelopment and gene regulation
Krumm et al. (2015) [126]	2377 families (2377 ASD probands, 1786 control siblings)	Exome sequencing	2.4-fold excess of de novo CNVs (deletions) were observed in ASD-affected relative to unaffected siblings, with CNVs in probands being significantly larger on average and enriched in variation-intolerant genes
Sanders et al. (2015) [127]	2591 families (2100 quads, 491 trios)	Genome-wide microarray analysis	The frequency, average size, and genic content of de novo CNVs was significantly higher among ASD cases relative to unaffected siblings
Leppa et al. (2016) [20]	1532 families (343 simplex, 1189 multiplex)	Genome-wide microarray analysis	Rare CNVs, including de novo events, were significantly more frequent among ASD-affected compared to unaffected siblings, as well as in simplex versus multiplex families, suggesting a higher burden of de novo variation in the genetic aetiology of sporadic ASD
Yuen et al. (2016) [40]	200 ASD trios^b^	Genome sequencing	Relative to controls, ASD probands demonstrated enrichment of deleterious de novo mutations in non-coding expression control regions (5ʹ/3ʹUTR), splice sites predictive of exon skipping, and DNase I hypersensitivity regions
Turner et al. (2017) [128]	516 ASD quads^b^	Exome sequencing	Significant enrichment for deleterious missense de novo SNVs/indels, promoter, and enhancer mutations were detected among ASD-affected relative to unaffected siblings
An et al. (2018) [129]	1902 ASD quads^b^	Genome sequencing	De novo risk scores were found to significantly predict ASD case status when localised to promoter regions characterised by evolutionary and functional signatures

*SNV* single nucleotide variant, *OR* odds ratio.

^a^Parental data also sampled and sequenced for each family and/or trio/quad.

^b^Familial trios (two parent + child) and quads (two parents + ≥2 children) consisted of one affected child only, unless specified as multiplex.

Environmental exposures that are classically attributed as salient risk factors for ASD and other neurodevelopmental disorders could represent a catalyst for deleterious de novo variation, with several disorder-associated agents having significant mutagenic and genotoxic potential [24]. However, although the neurotoxicity and teratogenicity conferred by these toxicants is well established [25], their potential role in the genesis of de novo mutations of relevance to ASD has received little attention. Toxicogenomic analyses suggest disorder-associated exposures may perturb known ASD susceptibility genes through mutagenetic chemical-gene interactions [26], however a paucity of evidence limits the current conceptualisation of this relationship to very few exposures (see Table 2). Moreover, in the last decade only one article has addressed the likely contribution of such agents to the de novo burden of ASD [24], pre-dating advances in next generation sequencing which have since contributed to a more comprehensive understanding of the significance of de novo variants to ASD. This review combines epidemiological data with evidence derived from assays of mutagenicity (i.e., in vivo and in vitro) to assess the plausibility of environmental exposures as sources of de novo ASD-associated genetic events. Elucidating the role of these agents in eliciting mutations will assist to delineate the basis of aetiological risk associated with non-familial forms of ASD. It may also encourage primary health interventions aimed at reducing the negative impact of environmental exposures on ASD risk.

**Table 2:** Environmental exposures associated with elevated ASD risk and evidence of their potential mode of mutagenicity/genotoxicity in vivo and/or in vitro.

Exposure	Link to ASD	Evidence of mutagenicity/genotoxicity
Direct interaction with genetic material		
Air pollutants		
1,4-dioxane	Average annual concentration of 1,4-dioxane at birth is associated with increased risk of ASD diagnosis (OR = 2.87) [28]	Oral administration of high-dose 1,4-dioxane significantly increases nucleotide transversions [130] and clastogenicity [131] in mammalian hepatic tissue in vivo
Acetaldehyde	Maternal exposure to acetaldehyde during pregnancy is associated with increased risk of ASD (OR = 1.20) [27]	Chronic inhalation of acetaldehyde at concentrations typical of ambient air in large cities results in the formation of DNA adducts in mammalian models [132]
Benzene	Maternal exposure to benzene during pregnancy is associated with increased risk of ASD (benzene OR = 1.46; ethylbenzene OR = 1.48) [27]	Occupational exposure to benzene induces increased DNA strand breaks in leucocytes [58], and is associated with aneuploidy of sex chromosomes [60] and chromosomal aberrations in sperm [59]
Diesel particulate matter	Elevated concentration of diesel particulate matter in ambient air at birth residence is associated with increased risk of ASD (OR = 1.44–2.0) [66, 67]	Occupational exposure to diesel particulate matter is associated with increased DNA strand breaks, nuclear buds, micronuclei formation, and nucleoplasmic bridges in lymphocytes [64, 133], and chromosomal aberrations including karyorrhexis, karyolysis, and chromatin condensation in buccal cells [64]
Vinyl chloride	Birth residence in census tracts with elevated vinyl chloride air pollution is associated with increased risk of ASD (OR = 1.75) [67]	Occupational exposure to vinyl chloride increases the frequency of chromosomal damage and aberrations, micronuclei formation, and DNA strand breaks in peripheral blood lymphocytes [65]
Heavy metals		
Aluminium	Trends in the use of aluminium adjuvants in vaccinations positively correlates with increases in ASD prevalence in the United States [17]	Prenatal exposure to aluminium in second and third trimester is associated with increased mitochondrial DNA copy number [134]; exposure to aluminium acetate induces chromosomal aberrations in mammalian hepatic cells in vivo [135]
Cadmium	Birth residence in census tracts with elevated cadmium air pollution is associated with increased risk of ASD (OR = 1.54) [67]	Exposure to cadmium results in increased DNA strand breaks in human hepatic and colorectal cells,[136] and demonstrates clastogenicity in mammalian lymphoblasts in vitro [137]
Herbicides		
Dioxins	Paternal exposure to dioxins during the Vietnam War is associated with higher rates of ASD in second generation offspring born abroad [138]	Increased de novo mutation rates have been reported among offspring born to fathers with high blood serum levels of dioxin [139, 140]
Organophosphates^a^	Maternal exposure to organophosphates in second (OR = 3.3) and third (OR = 2.0) trimester is associated with increased risk of ASD [52]; exposure to organophosphate congener chlorpyrifos in utero has been shown to elicit ASD-like social behaviours in mice models [141]	Organophosphate congener chlorpyrifos is associated with chromosome loss and missegregation in human lymphocytes [142], and loss of chromosome stability in sperm cells in vitro [143]. ^a^Exposure to chlorpyrifos in human foetal hepatic cells has been shown to induce DSBs and gene rearrangements in ASD susceptibility gene *KMT2A* [53]
Parental health factors		
Age	Increasing parental age at the time of conception is associated with increased risk of ASD (maternal OR = 1.41; paternal OR = 1.55) [33]	Exon [12, 144] and genome [43] sequencing data of ASD probands indicates paternal and maternal age each predict the number of de novo event per offspring
Pre- and post-conceptual drug use		
Antidepressant medication	Maternal use of any class of antidepressant in first trimester pregnancy (adjusted OR = 1.09–2.37) [30] and paternal selective serotonin reuptake inhibitor (SSRI) use prior to conception [145] is associated with increased risk of ASD in offspring	SSRI administration is associated with dose-dependent increases in micronucleus frequency and DNA strand breaks in peripheral blood lymphocytes [146], sister chromatid exchanges in sperm cells [147], and oxidative DNA damage in male germline cells in mammalian models in vivo [148]
Cocaine	Prenatal exposure to cocaine is tenuously associated with increased risk of ASD in childhood [32]	Frequent users of crack cocaine demonstrate increased micronuclei and nuclear buds in peripheral blood cells relative to non-users [149], and acute exposure to crack cocaine has been shown to induce DNA strand breaks in brain and peripheral blood cells in mammalian models in vivo [150]
Interference of endogenous DNA repair responses		
*See ‘*Multimodal effects’		
Oxidative DNA damage		
Air pollutants		
1,3-butadiene^a^	Maternal exposure to 1,3-butadiene during pregnancy (OR = 1.59) [27] and elevated concentration in ambient air at birth residence (OR = 1.57) [66] is associated with increased risk of ASD	Oxidative metabolites of 1,3-butadiene induce DNA-DNA crosslink adducts in mammalian models in vivo [151]; occupational exposure to 1,3-butadiene has been shown to increase urinary concentrations of oxidative stress markers of ROS-induced DNA damage [83]. ^a^Chronic inhalation of 1,3-butadiene over a 1-2 year period has been observed to elevate the incidence of point mutations in ASD susceptibility gene *HRAS* in gastric cells in mice in vivo [87]
Dibenzofurans	Average annual concentration of dibenzofurans at birth is associated with increased risk of ASD (OR = 2.53) [28]	Oxidative metabolites of dibenzofurans generate ROS in human hepatic cells in vitro [152]
Ethylene dichloride	Elevated concentration of ethylene dichloride in ambient air at birth residence is associated with increased risk of ASD (OR = 2.14) [66]	Oxidative metabolites of ethylene dichloride cause DNA adduct formation, gene mutations, and chromosomal aberrations in human cells in vivo and in vitro [153]
Styrene	Exposure to styrene in utero (OR = 1.8) [154] and maternal residence in regions with elevated chromium air pollution during pregnancy (OR = 2.04) [155] are associated with increased risk of ASD	Occupational exposure to styrene induces oxidative DNA damage in buccal cells [80] and DNA stand breaks in germline cells [90]
Tetrachloro-ethylene	Maternal exposure to tetrachloroethylene during pregnancy is associated with increased risk of ASD (OR = 1.40) [27]	Occupational exposure to tetrachloroethylene has been shown to induce DNA stand breakage [156] and acentric fragmentation [89] in peripheral blood lymphocytes. Tetrachloroethylene shares several oxidative metabolites with trichloroethylene
Trichloroethylene	Maternal exposure to trichloroethylene during pregnancy (OR = 1.14) [27] and birth residence in census tracts with elevated air pollution concentration (OR = 1.47) [67] are associated with increased risk of ASD	Exposure to trichloroethylene elicits a dose-response increase in ROS-induced markers of DNA double-stranded breaks in human hepatic cells [157] in vitro; maternal exposure to trichloroethylene during pregnancy is associated with increased levels of placental oxidative stress markers in mammalian models in vivo [158]
Heavy metals		
Beryllium	Elevated concentration of beryllium compounds in ambient air at birth residence is associated with increased risk of ASD (OR = 1.77) [66]	Maternal exposure to beryllium during pregnancy increases urinary markers of oxidative stress and DNA damage repair [88]; oral administration of beryllium chloride increases the frequency of micronuclei formation, DNA strand breaks, and ROS generation, and decreased expression of DNA repair genes [70], and is associated with dose-dependent increases in chromosomal aberrations in spermatocytes in mammalian models in vivo [159]
Chromium	Maternal residence in regions with elevated chromium air pollution during pregnancy is associated with increased risk of ASD (OR = 1.52) [155]	Occupational exposure to chromium increases oxidative stress response markers and DNA strand breaks in whole blood samples [160]; exposure to chromium and chromium metabolites in vitro has been demonstrated to elicit intracellular ROS generation in human neuronal analogues [161]
Lead	Proximity to industrial facilities with air lead [162], birth residence in census tracts with elevated levels of lead (OR = 1.57) [66], and maternal exposure to lead during pregnancy (OR = 1.49) [27] are associated with increased prevalence of ASD; exposure to lead in utero has been shown to elicit social-communicative dysfunction in mice models [163]	Occupational and residential exposure to lead are associated with increased DNA strand breaks in peripheral blood lymphocytes [85] and buccal cells [84]; and occupational exposure to lead is associated with elevated oxidative stress [84] and reduced cellular antioxidant activity [79]. Maternal exposure to lead during pregnancy increases oxidative stress in the developing foetus in mammalian models [164]
Nickel^a^	Birth residence in census tracts with elevated nickel air pollution is associated with increased risk of ASD (OR = 1.46-1.65) [66, 67]	Exposure to nickel in vitro has been associated with increased ROS generation and DNA strand breaks in human neuronal analogues [165] and human lymphocytes [166]. ^a^Exposure to nickel sulphide in vitro has been shown to increase mutagenesis in mRNA transcripts of ASD susceptibility gene *FHIT* in mammalian bronchial epithelial cells [167]
Herbicides		
Pyrethroids	Maternal proximity to pyrethroid insecticide applications in the pre-conception and/or third trimester period of pregnancy is associated with increased risk of ASD (OR = 1.7-2.3) [52]	Oral administration of pyrethroid congener fenpropathrin results in increased oxidative stress and DNA strand breaks in mammalian sperm cells in vivo [168]; in vitro exposure to pyrethroid congeners cyfluthrin, alpha-cypermethrin, and beta-cypermethrin elicits dose-dependent increases in ROS generation in human neuronal analogues [169]
Industrial chemicals		
Polybrominated Diphenyl Ethers	Trends in polybrominated diphenyl ether application positively correlates with increases in ASD prevalence in the United States [17]; maternal blood serum concentrations of polybrominated diphenyl ether congeners during pregnancy is associated with increases in idiosyncratic ASD behaviours in offspring [170]	Exposure to polybrominated diphenyl ether congeners results in increased oxidative stress in mammalian testicular tissue in vivo and increased ROS activity and expression of DNA repair genes in mammalian sperm cells in vitro [98]; high-dose concentrations of polybrominated diphenyl ether congeners elicit ROS generation and disrupted antioxidant status in human fibroblast cells in vitro [99]
Parental health factors		
Maternal diabetes	Pre-existing maternal diagnosis of type II diabetes (HR = 1.33) and development of gestational diabetes mellitus during pregnancy (HR = 1.42) [35], as well as maternal pre-pregnancy obesity and diabetes (HR = 3.91) and comorbid obesity and gestational diabetes (HR = 3.04) [34] increase the risk of ASD in offspring	Maternal diabetes during pregnancy increases the frequency of oxidative DNA double-strand breaks and DNA repair responses in mammalian embryos in vivo and mammalian neural stem cells in vitro [171]
Maternal iron deficiency	Maternal iron deficiency and anaemia during pregnancy increases the risk of ASD [172, 173]	Maternal iron deficiency during pregnancy increases oxidative stress in the placental tissue and developing foetus in mammalian models [174]; iron deficiency in humans is linked to increased biomarkers of oxidative stress and reduced antioxidant enzyme activity in peripheral blood [175]
Pre- and post-conceptual drug use		
Paracetamol (Acetaminophen)	Meta-analytical review indicates maternal use of paracetamol during pregnancy increases risk of ASD in offspring (RR = 1.19) [29], and elevated biomarkers of paracetamol metabolites in umbilical cord plasma is associated with childhood ASD (OR = 2.14–3.62) [115]	Oral administration of therapeutic doses of paracetamol induces oxidative stress-related gene expression in peripheral blood in humans in vivo [176]; elevated concentration of urinary paracetamol is associated with increased DNA fragmentation in human sperm in vivo [177]
Thalidomide	Prenatal exposure to thalidomide is associated with increased risk of ASD in childhood [32]; administration of thalidomide during pregnancy has been shown to elicit idiosyncratic ASD behaviours and nonexploratory movement in mammalian offspring [178]	Exposure to thalidomide elicits oxidative DNA damage in mammalian embryonic cells in vitro [179]; administration of oxidative dihydroxy thalidomide metabolite has been shown to induce DNA strand breaks and elevated intracellular ROS in human embryonic, hepatic, and endothelial cells in vitro [180]
Multimodal effects		
Air pollutants		
Formaldehyde	Maternal exposure to formaldehyde during pregnancy is associated with increased risk of ASD (OR = 1.34) [27]	Long-term exposure to low-dose formaldehyde reduces antioxidant capacity in testicular tissue [96] and causes changes in oxidative stress enzymes in ovaries in mammalian models in vivo [97]; occupational exposure to formaldehyde increases cytogenic alterations in lymphocytes [181], and low-dose exposure reduces nucleotide excision repair in human cells in vitro and mammalian systems in vivo [72]
Quinoline	Exposure to quinoline in utero is associated with an increased risk of ASD (OR = 1.40) [154]	Quinoline exposure demonstrates mutagenicity through DNA adduct formation, unscheduled DNA synthesis, and chromosomal aberrations in mammalian cells in vitro [182]
Heavy metals		
Arsenic	Proximity to industrial facilities with air arsenic is associated with increased prevalence of ASD [162]	Occupational and residential exposure to arsenic are associated with increased DNA strand breaks in peripheral blood lymphocytes [85, 86]; in vitro exposure to arsenic has been demonstrated to elicit ROS-dependent mitochondrial DNA damage in mammalian oocytes [183]
Copper	Maternal exposure to copper compounds during pregnancy is associated with increased risk of ASD (OR = 1.09) [27]	Maternal exposure to copper during pregnancy increases urinary markers of oxidative stress and DNA damage repair [88]; in vitro exposure to copper has been shown to elicit structural changes in chromatin confirmation and DNA denaturation in cortical tissue [184], and increases the frequency of micronuclei, nucleoplasmic bridges, and nuclear buds in human lymphocytes [185]
Manganese	Elevated concentration of manganese in ambient air at birth residence is associated with increased risk of ASD (OR = 1.54) [66]; exposure to manganese in utero elicits behavioural idiosyncrasies in preservative/impulsive and social dominance behaviours similar to ASD in mammalian models [163]	Maternal exposure to manganese during pregnancy increases urinary markers of ROS generation [88]; in vitro exposure to manganese increases the frequency of micronuclei, nucleoplasmic bridges, and nuclear buds in human lymphocytes [185]
Mercury	Proximity to industrial facilities with air mercury [162] and birth residence in census tracts with elevated levels of mercury (OR = 1.92) [67] are associated with increased prevalence of ASD	Occupational exposure to mercury is associated with karyolysis in peripheral blood lymphocytes [86]; prenatal exposure to mercury in utero disrupts development of the glutathione antioxidant system, increasing susceptibility to pro-oxidative damage in offspring [186]; mercury exposure in vitro increases biomarkers of oxidative stress and ROS generation in human glial [187] and microglial cells [188]
Herbicides		
Glyphosates	Trends in glyphosate application positively correlates with increases in ASD prevalence in the United States [17]	Exposure to glyphosates at or below the agreed acceptable daily limit for human health induces cytogenic changes in human hepatic cells [189], and oxidative stress and double-stranded DNA breaks in mammalian oocytes [190] in vitro
Organochlorines	Maternal residence proximal to agricultural applications of organochlorine pesticides during pregnancy increases the risk of ASD in offspring (OR = 6.1) [191]; maternal blood serum concentrations of organochlorine congeners during pregnancy is associated with increased idiosyncratic ASD behaviours in offspring in early childhood [170]	Exposure to organochlorine congener endosulfan is associated with ROS-induced double-stranded DNA breaks in human leucocytes in vitro and mammalian testicular tissue in vivo [192], as well as perturbed DNA repair in human leucocytes in vitro [193] and mammalian cells in vivo [192]
Industrial chemicals		
Phthalates	Prenatal exposure to phthalate significantly increases risk of ASD and ASD traits at age 4 (adjusted OR = 1.65 per SD unit increase in total phthalate) [194]	Occupational exposure to phthalate congeners induces oxidative stress, antioxidant enzyme activity, and ROS-dependent DNA damage in peripheral blood [81] and changes in mitochondrial DNA copy numbers in sperm cells [82]; phthalate congener dibutyl phthalate has been shown to elicit double-stranded DNA breaks in mammalian neural progenitor cells in vitro [195] and reduce expression of DNA repair genes in mammalian mouse ovaries in vivo [69]
Parental health factors		
Maternal folate supplementation	Moderate periconceptual [37, 196] and first trimester [37, 38] folate supplementation reduces ASD risk in offspring	Decreased folate concentrations in blood serum is associated with increased oxidative stress-dependent DNA strand breaks in at-risk manufacture workers [197]; diet-induced folate deprivation inhibits expression of base excision repair genes in response to oxidative DNA damage in mammalian models in vivo [198]
Maternal vitamin status	Multivitamin supplementation (vitamin A, B, C and D) pre- and during pregnancy is associated with reduced risk of ASD [37, 93]; low serum levels of vitamin D [36] and maternal vitamin D deficiency during pregnancy [199] increases ASD risk in offspring	Maternal vitamin D deficiency in human mothers during pregnancy increases the frequency of micronuclei in umbilical cord blood at birth [200]; vitamin D supplementation protects against oxidative DNA damage in mammalian neuronal cells in vitro [94]
Pre- and post-conceptual drug use		
Cannabis	TCH exposure affects ASD-associated genes in human induced pluripotent cell-derived neurons in vitro [31]	Oral administration of TCH at concentrations equivalent to illicit preparations induces DNA strand breaks and oxidative stress responses in mammalian brain tissue in vivo [201]; exposure to low-dose concentrations of cannabidiol and cannabidivarin elicits DNA strand breaks, oxidation of DNA nucleotides, and chromosomal aberrations in human hepatic cells in vitro [202]
Opioids	Maternal preconceptual prescription of opioid medication is associated with elevated risk of ASD in offspring (OR = 2.43) [203]	Prolonged use of methadone induces DNA strand breaks in peripheral blood lymphocytes [204]; low- and high-dose exposure to codeine induces oxidative sperm DNA fragmentation in mammalian models in vivo [205]; exposure to morphine significantly alters the expression of genes associated with nucleotide excision repair, non-homologous end-joining, and base excision repair in human mammary and neuronal analogue cells in vitro [71]
Valproate	Maternal valproate use during pregnancy is associated with increased risk of ASD in offspring (adjusted HR = 2.9), even when controlling for maternal epilepsy status (adjusted HR = 1.7) [75]; administration of valproic acid during pregnancy has been shown to elicit idiosyncratic ASD behaviours, nonexploratory movement [178] and socio-communicative deficits [74] in mammalian offspring; juvenile non-human primates exposed to valproic acid in utero demonstrate impaired social interaction, non-social eye-gaze preference and stereotypic behaviours stereotypical of ASD [206]	Exposure to valproic acid in utero decreases antioxidant enzyme activity [207], elicits increased intrachromosomal recombination [73] and DNA strand breaks [74], and impairs DNA damage repair responses in mammalian embryos in vivo [73, 74]. Impairment of DNA damage repair responses have been positively associated with deficits in social-communicative behaviours in valproic acid-exposed mammalian offspring [74]

See Supplementary Information for details pertaining to the search terms and search strategy.

*HR* hazards ratio, *OR* odds ratio, *RR* risk ratio.

^a^Indicates exposure has been demonstrated to selectively target ASD susceptibility gene.

## Mutagenicity and genotoxicity of early-exposure to established ASD toxicants

Although there is no support for a causal role for any single environmental risk factor in the onset of ASD, several pre-conceptual and prenatal environmental agents have been associated with increased risk of ASD and ASD-like symptoms. The most widely cited sources of environmental risk include exposure to toxins and heavy metals in utero [27], birth and maternal residency proximal to sources of ambient air pollution [28], prescription [29, 30] and illicit [31, 32] drug use during pregnancy, and parental health factors including age [33], maternal obesity and diabetes [34, 35], and pre- and peri-gestational vitamin status [36–38].

Very few direct assessments of the potential role of exposures in eliciting de novo mutations in ASD exist. However, significant observations have surfaced from cohort studies as to how parental age may contribute to spontaneous mutations associated with the disorder. Advanced paternal [39–41] and, more recently, maternal age [12, 33] at the time of conception have been demonstrated to confer an elevated risk of ASD development. To this end, age-associated accumulation of gametal DNA damage and failure of intrinsic repair mechanisms to excise acquired errors act as potent inducers of ASD-associated de novo risk. It is hypothesised that lifelong spermatogenesis affords recurrent opportunities for DNA damage and mis-repair in the generation of mature sperm [42], whereas prolonged meiotic arrest enhances the likelihood of damage-induced lesions accruing in the genetic structures of primary oocytes [43]. It is important to note that de novo risk persists beyond insemination, with rapid mitotic events during early embryogenesis elevating the risk of DNA damage and mutational events. Further, there is mounting evidence of differential mutational rates and mechanisms in postzygotic somatic mosaicism [44, 45], which is a current area of much-needed additional research.

**Fig. 2:**
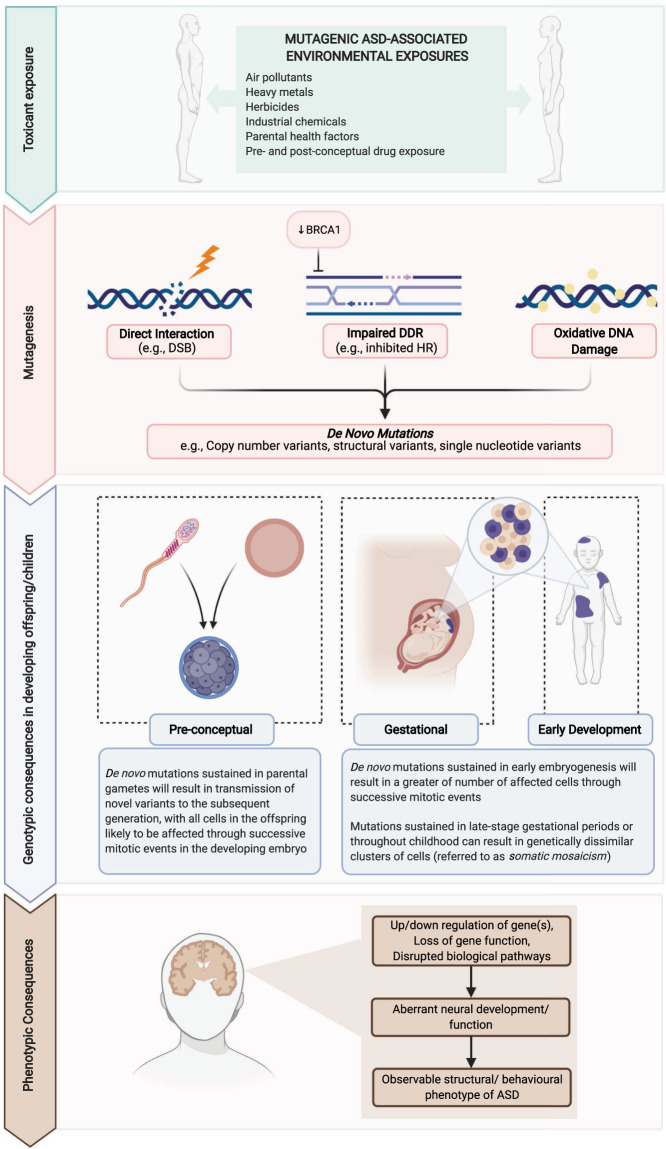
Diagrammatic representation of the impact of environmental factors on genomes within parental germlines and offspring. ASD-associated toxicants (e.g., herbicides, heavy metals) can induce de novo mutations in parental germline cells which may be transmitted to offspring in the subsequent generation. For example, agent-induced double stranded breaks (DSBs) and impaired BRCA1-directed homologous recombination (HR) DNA damage response (DDR) can elicit de novo mutations and hamper their repair. Offspring may also acquire agent-induced mutations at later stages of development, resulting in somatic mosaicism. Gene(s) impacted by these processes can lead to aberrant neural development and functioning, contributing to the onset of ASD. Figure created with BioRender.com.

Table 2 provides an extensive audit of current environmental factors identified via epidemiological studies as salient influencers of ASD risk. Although less thoroughly researched than the association with parental age, many exhibit evidence of mutagenicity and genotoxicity in human and relevant mammalian models. Broadly, these exposures can be observed to contribute to genomic alterations through one of three potential modes of action: (1) direct interaction with genetic material, both at the nucleic and chromosomal level; (2) interference with endogenous DNA repair; and (3) indirect DNA damage elicited through exposure-induced oxidative stress (see Fig. 2). Each of these processes has the capability to elicit genotypic abnormalities in biological systems and are, therefore, important to consider in relation to the de novo mutational burden of ASD.

### Direct interaction with genetic material

Several environmental agents exert their mutagenic and/or genotoxic potential by altering the DNA structure. Such mutations are primarily the consequence of toxicant-induced double-stranded breaks (DSBs): lesions to the DNA duplex which result in loss of stability and integrity in both strands of the nucleic acid helix. DSB repair is readily enacted by endogenous systems of *non-homologous end-joining* (NHEJ; i.e., ligation of two strands of damaged DNA) and *homologous recombination* (HR; i.e., template-dependent repair) [46], however aberrations may occur if the capacity for repair is exceeded by the degree of damage, or if repair-directed ligation results in erroneous rearrangement of DNA fragments [47]. The structural abnormalities that arise from un- or mis-repaired DSBs act greatly contribute to genomic instability, conferring risk for an array of genomic anomalies including nucleotide insertions and deletions (collectively referred to as *indels*) [48], gross chromosomal rearrangements [49], and fragmentation of chromosomes or chromatids [50], all of which can be sustained through multiple cycles of mitotic division. If such damage were to occur pre-gestationally in maternal or paternal gametes, or post-conceptually in early stages of embryogenesis, it is plausible that this could lead to presentation of novel genomic variation in offspring that is absent in either parental genome [51].

ASD-associated toxicants including chlorpyrifos, benzene, diesel particulate matter, and vinyl chloride induce various cellular phenotypes of genomic instability indicative of DSB DNA damage. Chlorpyrifos is an organophosphate congener widely applied in residential pest control, the use of which has been shown to possibly increase the incidence of ASD among prenatally exposed children [52]. Critically, chlorpyrifos have been shown to elicit DSBs and genetic rearrangements in the well-established ASD susceptibility gene *KMT2A* [53], a transcriptional coactivator gene repeatedly reported to harbour loss-of-function de novo variants among individuals with ASD [12, 54–57]. Conversely, benzene is an organic chemical compound widely applied in the industrial manufacture of plastics, and is present in petrochemicals including gasoline. Occupational exposure to benzene and benzene metabolites has been repeatedly associated with DSBs in intracellular nuclei [58], and confers abnormal morphology [59] and segregation of sex chromosomes in mature sperm [60]. Chromosomal instability elicited via DSBs increases the incidence of missegregation through disrupted genomic and protein integrity during mitotic division [61], potentiating the uneven distribution of genetic material in daughter cells. X/Y chromosome aneuploidy is frequently observed in clinical ASD cohorts [62], hence atypical inheritance of sex-linked genes (e.g., *FMR1, NLGN3/4*) orchestrated through toxicant-induced DSBs and DNA missegregation in parental gametes is a feasible mechanism of ASD risk.

Although potentially less deleterious than entire chromosomal losses or gains, micronuclei are important structural anomalies indicative of genomic instability and DSB mis-repair in cells exposed to harmful genotoxins [63]. The incidence of micronuclei and related extra-nuclear structures positively correlate with occupational exposure to diesel particulate matter among mechanics [64] and vinyl chloride in thermoplastic manufacturing facilities [65], both of which are considered salient toxicants in the aetiology of ASD [66, 67]. These extra-nuclear bodies can originate through the enveloping of acentric chromosome and chromatid fragments formed as consequence of asymmetrical DSB repair [47, 63]. Compartmentalised fragments of damaged DNA within these structures are vulnerable to acquisition of mutations through repeated cycles of defective replication and impaired recruitment of damage response pathways [68], and can reincorporate into the primary nucleus following breakdown of the nuclear membrane during mitosis. Daughters of micronucleated parent cells are therefore likely to harbour an array of genomic rearrangements including indels, chromosomal translocations, and copy-number variation in gene-coding sequences [61], all of which represent core features of the molecular architecture of ASD.

### Interference of endogenous DNA repair responses

Toxicants capable of eliciting damage to the DNA structure are ubiquitous in the environment, however the harmful consequences of these are largely mitigated through rapid-activation of DNA damage response (DDR) pathways. DDRs are responsible for the detection and amelioration of aberrations in the genomic structure, and if necessary, can initiate apoptotic events to prevent transmission of lesioned genetic materials through DNA replication and mitotic division [69]. Several DDR pathways are perturbed through exposure to ASD-associated genotoxins, limiting the repair system’s ability to rectify acquired mutations and, by proxy, their affiliated biological consequences. Specifically, these include: the aforementioned NHEJ and HR pathways, which are responsible for extruding acquired DSBs; *mismatch repair* (MMR), which is necessary to amend errors in base-to-base alignment in replicated DNA; and *base-* (BER) and *nucleotide excision repair* (NER), which are enacted to correct single-base and bulky DNA lesions, respectively [46]. In many cases, mutations arise through exposure-induced delay or impaired recruitment of the machinery required to initiate DDR, hampering the overall DNA repair efficiency. The consequence of ineffective or inhibited repair responses by these systems are likely to coincide a vast array of genetic anomalies, ranging from single base-pairs to entire gene-encoding sequences, conferring significant and arguably the most pronounced risk to the acquisition of deleterious disorder-associated mutations.

The most common mode of DDR interference exerted by ASD-associated exposures is suppression of genes whose products are necessary to detect, excise, or amend lesions in the nucleotide structure. Several genes encoding proteins responsible for the identification of genomic anomalies are markedly downregulated in the NER (e.g., *XPC*) [69], BER (e.g., *APEX1*) [70], MMR (e.g., *MSH3, MSH6*) [69], and NHEJ (e.g., *KU70/80*) [71] pathways following exposure to ASD toxicants, suggesting these exposures may impair the ability of DDR mechanisms to effectively respond to damage in the DNA structure or sequence. Activation of the DDR pathways hinges on the capacity of the cell to detect aberrations in the genetic code, hence interference in the expression of these may cause delay or complete arrest in the damage-directed repair response. Although less frequently observed, genes encoding products required to effectively excise detected aberrations also demonstrate dysregulation following exposure to disorder-associated environmental agents, specifically within the NER response to induced DNA adducts (e.g., *XPG*) [72]. In such instances, acquired mutations remain unextruded despite appropriate activation of the DDR, precluding the repair response and permitting DNA damage and, thereby, de novo variation to persist.

Critically, exposure-induced dysregulation of genes required in the assembly and incision of corrected DNA sequences can be observed in both the HR (e.g., *RAD51*) [73, 74] and NHEJ (e.g., *XRCC4*) [71] DSB response pathways, suggesting ASD-associated toxicants are not only capable of inducing DSB lesions, but also inhibiting their effective repair. This represents a salient risk for the acquisition of de novo mutations, as un- or mis-repaired DSBs exert potent mutagenic potential as previously discussed (see section *Direct interaction with genetic material)*. Remarkably, suppressed expression of HR repair genes following exposure to valproate, an anticonvulsant drug associated with elevated ASD risk [75], has been linked to idiosyncratic deficits in social-communicative behaviours in mammalian models [74], suggestive of a potentially causative relationship between impaired DDR and development of the disorder phenotype. Although it is plausible that this relationship could be mediated, at least in part, by accumulation of genetic aberrations through failure to appropriately repair acquired lesions, experimental confirmation is required.

Relative to HR, NHEJ DSB repair confers greater risk of acquiring structural anomalies through its ability to ligate strands of damaged DNA irrespective of homology. The unspecific nature of this mechanism allows tethering of mismatched or significantly mutilated termini, increasing the likelihood of gross chromosomal rearrangements or loss of genetic material [68]. To enact HR following lesion acquisition, an intact sister chromatid is required to act as a template for replication and synthesis of the damaged genetic material, resulting in high-fidelity repair. Initiation of HR is largely dependent on the breast cancer type 1 susceptibility protein, BRCA1, which can elicit DNA resection to inhibit NHEJ and trigger homology-directed repair [76]. Susceptibility to mutagenic events in early embryogenesis necessitates accurate DDR, hence HR is implemented as the preferred mechanism for DSB repair at this stage of development [77]. Exposure to ASD toxicants including valproate and phthalate, the latter a common plasticiser, downregulate *BRCA1* in mammalian ovarian cells [69, 73] thereby reducing availability of the BRCA1 protein for the purposes of DNA resection. This increases reliance of the developing cellular system on the error-prone NHEJ DDR to ameliorate acquired DSBs, elevating the risk of, and frequency at which, the foetus will sustain de novo mutations.

### Oxidative DNA damage

Generation of oxidative stress and reactive oxidative species (ROS) are important components of cellular metabolism, however, can become genotoxic when produced en masse in response to harmful environmental toxicants. The capacity for this otherwise non-pathogenic process to elicit DNA damage is due to the highly reactive hydroxyl radical (^•^OH). ^•^OH forms double bonds with bases in the nucleic structure and abstracts hydrogen atoms from both the carbon-hydrogen bonds of 2’-deoxyribose and the methyl groups of thymine nucleotides [78]. This can elicit several genomic changes, including base-specific modifications, DNA-DNA and DNA-protein crosslinks, and both single- and DSBs [79]. Several classes of ASD-associated exposures elevate ^•^OH ROS production and redox cycling across a range of mammalian and human tissue, suggestive of potentially ubiquitous consequences to the biological system.

Occupational and residential exposures are among the primary sources of oxidative DNA damage for several key ASD-associated toxicants. Prolonged contact with ROS-inducing agents increases the intracellular burden of oxidation, permitting accrual of genotoxic and cytotoxic lesions as consequence of persistent generation of ^•^OH radicals. Among those aetiologically relevant exposures, the contaminants individuals are likely to interact with chronically – either through workplace activities or residential pollutants—include oxidative chemicals used in the production of synthetic materials (e.g., phthalates, styrene) [80–82], components of petroleum exhaust (e.g., 1,3-butadiene) [83], and toxic chemicals such as lead [84, 85] and mercury [86] utilised in industrial manufacturing processes. Of note, the oxidative DNA damage associative of chronic inhalation of 1,3-butadiene has been demonstrated to selectively target *HRAS*, a high-confidence ASD susceptibility gene, resulting in elevated point mutation accrual in affected cells [87]. Although typically assessed through urinary markers of oxidative DNA damage (i.e., 8-hydroxy-2′-deoxyguanosine, or 8-OHdG) [83, 88] or conveniently acquired biologicals (e.g., blood samples, buccal cells) [80, 89], occupational exposure to chemicals including phthalates [82] and styrene [90] have been directly observed to induce DNA aberration in gametes, supporting the capacity for ROS-induced lesions to localise to reproductive tissue. If viable, these mutated cells may result in genetic anomalies in subsequent offspring, thereby affording a mechanism for elicitation of non-familial genomic variability.

To counteract the pathological consequences of environmentally-induced oxidative stress, the biological system relies on a complex array of endogenous and exogeneous antioxidant defence mechanisms. These include metabolic antioxidant enzymes (e.g., glutathione peroxidase, catalase, superoxide dismutase), non-enzymatic proteins (e.g., lactoferrin), and scavengers of free radicals (e.g., iron) [91, 92]. Collectively, these constituents protect intracellular DNA and the broader cellular system from the damaging effects of excessive oxidative stress. It is therefore unsurprising that antioxidant deficiencies or interruption of their endogenous functions can indirectly contribute to the acquisition of genomic lesions if sustained over time. Maternal multivitamin supplementation during pregnancy has been shown to both reduce the incidence of ASD onset [37, 93] and buffer against oxidative DNA damage [94, 95], whereas ASD toxicants such as formaldehyde, a gaseous chemical used in the production of building materials, and flame retardant constituents including polybrominated diphenyl ethers disrupt antioxidant status in both gametal [96–98] and non-reproductive cells [99], potentiating oxidative imbalances and ROS-induced DNA damage in afflicted tissues.

### De novo risk persists beyond embryogenesis

Spontaneous genetic mutations can be acquired at any stage of the lifespan [44, 45], and although those preceding or in early embryogenesis elicit a greater number of cells harbouring genetic anomalies (see Fig. 2), exposure-induced mutations in later periods of pregnancy or childhood may perturb neurodevelopment if sustained in temporally-critical brain-expressed genes. Mutations elicited in the post-zygotic period are restricted to specific subsets of dividing somatic cells, resulting in genetically dissimilar assemblages of tissue within the biological system [100]. This phenomenon, referred to as *somatic mosaicism*, permits variable expression of genes across clusters of daughter cells with diverse parental lineages, creating irregularity in the availability and integrity of the encoded protein [101]. If harboured in neural tissue prior to scheduled periods of brain development, disruption of genes central to processes of proliferation, synaptogenesis, or synaptic pruning may result in aberrant or stunted neurological growth; a cornerstone feature of the disorder phenotype (see Fig. 2). The deleterious effects of localised genetic lesions in ASD have been evidenced in histopathological studies of post-mortem brain tissue, with atypical segments of neocortical architecture in the frontal and temporal regions arising as consequence of somatic mutations in a subset of ASD patients [102, 103].

The incidence of de novo mutations and their role in the underlying pathology of ASD beyond early development has received little research attention, however the mutational capacity of environmental factors represents a source of somatic mosaicism likely to influence neural maturation throughout childhood. Although in isolation these are unlikely to elicit symptomatic presentations of ASD or ASD-like behaviours, such mutations may play an influential role in augmenting the severity and persistence of symptoms for at-risk individuals, hence contributing to the functional impairment coinciding clinical diagnosis.

### Elevated genomic sensitivity to exposure-induced mutagenesis

Elevated genomic instability among ASD probands may enhance sensitivity to exposure-induced mutagenesis, increasing opportunity for mutation accrual and, by extension, de novo variation. Tandem repeat DNA motifs are highly liable genomic constituents prone to spontaneous somatic mutations [104], with increasing numbers of motif iterations (i.e., *expansions*) elevating the likelihood of novel genetic variation [105]. Constituting ~6% of the human genome, these units of repetitive DNA are known to contribute to molecular dysfunction across several complex clinical phenotypes, with recent analyses implicating repetitive DNA variants in the aetiology of ASD. Genome-wide interrogation of rare tandem repeat expansions has suggested that up to 2.6% of ASD risk may be explained by tandem expansions of repeat sequences enriched in exonic and splice sites across the genome, and often correlating with fragile sites [106]. In a separate study of a subset of the samples analysed by Trost and colleagues [106], the role of tandem repeat alterations in ASD was validated [107], further signalling that tandem repeats represent a significant component of the genetic aetiology of ASD. Critically, ASD-associated tandem repeat expansions were determined to be further expansions of large, inherited motifs, suggesting a transmission bias of these genomically unstable units among ASD probands [106, 108]. This preferential inheritance of mutationally liable DNA may therefore augment susceptibility to the mutagenic actions of environmental toxicants, likely increasing the incidence of putative de novo events across predominantly protein-coding and alternative transcription sites (we use the term *putative* due to the complexities in defining what is considered de novo in dynamic regions of the genome such as tandem repeats). The phenotypic consequences of such mutational events would vary depending on the putative gene affected, however given the elevated burden of de novo variation in loss-of-function intolerant regions among sporadic cases of ASD (see Table 1), the effects on clinical presentation are likely to be pronounced.

### Environmental agents and epigenetics

Harmful exposures in the pre- and post-pregnancy period may elicit non-mutational epigenetic changes to the expression of genes central to early development [109]. Epigenetic modifications provoke transformations in the conformational arrangement of the nuclear structure and modulate intracellular activity, regulating the capacity for molecular machinery to transcribe and translate encoded genetic materials without directly affecting the underlying DNA. Several toxicants implicated in the aetiology of ASD are known to disrupt epigenetic mechanisms, particularly processes of DNA methylation [110], across developmentally sensitive stages of neural differentiation, proliferation, and migration [111], indicating that these non-genomic gene-environment interactions are important determinants in the susceptibility profile of ASD. Research suggests, however, that the role of the epigenome in perturbing processes central to the pathophysiology of ASD may extend beyond dysregulated gene expression. Studies concerning the mechanisms of tumorigenesis, a pathology largely underpinned by mutation accrual and disrupted gene regulation, have discerned that aberrant functioning in key epigenetic regulators may be causative of de novo genetic events [112]. For example, methylation of cytosine residues in CpG dinucleotides has been associated with repair-resistant T:G nucleotide mismatches, elevating base-level mutation rates at CpG sites relative to other dinucleotides across the genome [113]. Furthermore, accumulation and persistence of de novo events has been demonstrated to coincide promoter hypermethylation of key cell signalling and DDR-associated genes [112], thereby contributing to de novo mutations through both elicitation and impaired rectification of novel DNA variants. Evidence implicating DNA methylation as a target of ASD-associated toxicants has been reviewed elsewhere [110], and provides compelling support for the physiological consequences these agents exert on the epigenome, suggestive of its potential role in elicitation of de novo variation.

### Limitations and future directions

The parallels between epidemiological trends and mutagenicity following exposure to ASD toxicants leads to intriguing hypotheses regarding the role of environmental agents in eliciting de novo mutations. Nonetheless, discerning causality in relation to the incidence of sporadic ASD remains challenging given the current state of the available research literature and therefore our review should support hypothesis building.

A key limitation in characterising the gene-environment relationship underscoring the de novo burden of ASD is the disparity in approaches from which heritable and non-heritable aetiological evidence is derived. On the one hand, identification of environmental agents that are likely to influence ASD development has depended on comparing the rates of ASD between exposed versus unexposed children from population cohorts (see Table 2). Since family-based designs are typically not employed within such studies, data is lacking both on the rate of de novo mutations in these samples and therefore the potential causative role of environmental exposures on de novo mutations. Clinic-referred samples, on the other hand, have afforded great insights into the contribution of rare, de novo mutations to the aetiology of ASD, but have rarely surveyed environmental risk as a modifier of this contribution, with the exception of parental age. As consequence, there is no informative estimate as to the proportion of de novo mutations among ASD probands which develop in response to environmental toxicant exposure. Moreover, although evidence supports a contribution of de novo mutational load in ASD at the level of specific genes and pathways, there is no overwhelming support for this effect genome-wide [21]. Thus, although we consider this line of inquiry to be of significant aetiological interest, we acknowledge the inherent limitations in trying to bridge these literatures.

A further limitation for the field is the absence of longitudinal evidence - human or otherwise - which simultaneously considers acquisition of non-familial genetic variation and ASD outcomes following pre- or post-conception toxicant exposure. The prognostic trajectory of ASD and other neurodevelopmental conditions has led to increasing interest and activity in establishing prospective birth cohorts to monitor environmental correlates of childhood disorder phenotypes (e.g., the Children of Nurses’ Health Study [66], Table 2). This has permitted the detrimental effects of developmental exposures to be observed in relation to symptomatic onset in later life, enhancing understanding of the non-heritable aetiology of ASD. Nevertheless, incorporation of the biological techniques necessary to detect and trace mutational events among such cohorts is limited, if not entirely absent. As such, this review has drawn upon broader evidence of exposures’ mutagenicity from in vivo and in vitro methodologies. Although the literature reviewed provides tantalizing insights into the ways in which environmental exposures might be linked to de novo mutational events, disorder-specific causation is lacking. To establish the role of disorder-relevant genotoxins in enhancing the de novo burden of ASD, both the genotypic and phenotypic consequences of parental/prenatal exposures must be assessed prospectively from birth in broader population cohorts. This will not only permit identification of novel genetic variation resulting from harmful toxicants, but allow the corollaries of acquired mutations on neurodevelopment to be traced across sensitive periods of childhood.

Identification of spontaneous de novo mutations relies on availability of the genetic information from each of the mother, father, and offspring, allowing intergenerational divergence in the DNA sequence to be distinguished from inherited allelic compositions [12]. Detection and assessment of acquired genomic abnormalities has traditionally relied on cytogenetic assays which, although capable of discerning significant aberrations in the chromosomal structure [101], cannot capture less conspicuous mutations known to contribute to the genetic aetiology of ASD. Current conceptualisations of the mutagenicity and genotoxicity intrinsic to several environmental toxicants has therefore been limited to their capacity to elicit large-scale malformations in the genomic structure, neglecting the subtler effects these exposures may exert on the genetic sequence. With the development of next generation sequencing (NGS) methods, however, the ability to identify nucleic acid deviations in as little as a single base pair in offspring relative to parental genomes is achievable, accurate, and increasingly affordable. NGS involves comprehensive assessment of the nucleic arrangement of either the entire genome (i.e., *whole genome sequencing*) or specifically gene-encoding segments of DNA (i.e., *whole exome sequencing*), allowing variation in the genomic code to be detected and compared between individual genomes. Such techniques have provided important insights into the rate of spontaneous genetic variation in cases of sporadic ASD, demonstrating the importance of genotypic variation to ASD development [19]. The advances in technology which spurred the advent of NGS have significantly reduced the costs associated with performing high-throughput sequencing, hence it is feasible to apply these to existing and emerging birth cohorts to assess the origin and frequency of de novo mutations among infants diagnosed with ASD in childhood. Such data would provide crucial insight into the genuine risks environmental toxicants pose to accentuating the genetic aetiology associated with ASD, enhancing our understanding of the mechanisms by which these agents contribute to the ASD phenotype.

Concerns for the harmful effects of prenatal exposure to environmental toxicants has led to a maternal bias in epidemiological studies assessing trends in ASD onset. Although such concerns are founded on well-established evidence for the disruptive consequences of exogenous agents on foetal development in utero [25], this focus has arguably undermined the significance of pre-conception paternal exposures. Relative to oocytes, spermatocytes appear to possess heightened sensitivity to genotoxic agents within the environment, putting male gametes at greater risk of acquiring and transmitting genomic lesions to the subsequent generation [91]. As previously discussed, recurrent spermatogenesis across the lifespan elevates the risk of defective DNA replication and repair in male relative to female gametes [43]. Should the testicular tissue from which spermatozoa divide acquire environmentally-induced genomic damage, as has been evinced following exposure to several known ASD toxicants (see Table 2), it is likely that resultant sperm cells will harbour genetic abnormalities. Germline susceptibility to environmental agents may be additionally mediated by the intrinsic ability of cells to effectively employ DDR following harmful exposures, the capacity for which is significantly compromised in sperm [114]. During spermatogenesis, the nuclear chromatin within the cell becomes highly condensed to improve motility for insemination. As consequence, the capacity to effectively excise and extrude genomic aberrations is limited, hence mutations in the genomic sequence may persist to fertilisation. Although there is evidence to support the capacity of the fused oocyte to perform post-fertilisation DDR of spermatic DNA, the frequency of de novo mutations of paternal origin in atypically developing offspring [33] suggests this repair response is imperfect. Given such sensitivity to mutagenic agents, assessing the incidence of ASD among offspring of fathers exposed to toxicants is essential to characterising the role of paternally acquired mutations in enhancing onset risk, further characterising the aetiological basis and more specifically the de novo origin of the disorder.

Notwithstanding these limitations, understanding the mutagenic mechanisms of environmental toxicants and, by proxy, their role in the elicitation of de novo risk variants, may inform public health initiatives to combat increasing rates of ASD within the community. Mutagenic exposures such as parental drug use and pre- and post-conceptual health factors offer targetable and inexpensive intervention opportunities which may assist to circumvent disorder-associated mutational events. For example, maternal folate and vitamin D supplementation prior to and during gestation has been shown to significantly reduce the rate of ASD among offspring [37]. In contrast, administration of substances such as paracetamol [29, 115] and antidepressant medications [30] during pregnancy is associated with increased risk of disorder onset in the subsequent generation. Addressing these modifiable factors may prove to be effective combatants to the de novo burden of ASD, warranting further research in the interest of public health. To this end, the advent and increased accessibility of novel molecular tools such as induced pluripotent stem cell (iPSCs)-derived neuronal lines has equipped researchers to evaluate agent-induced mutagenesis in clinically relevant tissue [116], facilitating the evaluation of the current hypothesis and its utility in informing primary intervention strategies. Recent advances in developing evidence-based list of genes relevant to autism and the neurobehavioral characteristics associated with it [117] will also enable more specific hypothesis testing in model systems, as well as epidemiological studies.

### Concluding remarks

The paradox of stable (or increasing) ASD rates in the general population despite reduced fecundity provides a compelling rationale for the contribution of de novo mutations to the genetic aetiology of ASD [12]. The potential for non-heritable factors such as environmental toxicants to elicit de novo events may afford an explanation for the underlying mutational trigger, leading to novel pathogenic mutationsin specific disorder-associated gene(s), thereby contributing to symptomatic onset. At both the pre- and post-gestational period, harmful environmental agents may induce genomic lesions through a myriad of genotoxic mechanisms, summarised within this review into direct, repair inhibition, and oxidative DNA damage induction. Acquired mutations in parental gametes or the developing embryo may potentiate the disorder phenotype if localised to genes salient in processes of general development or specific neurodevelopmental pathways, however this risk may persist throughout maturation through the phenomenon of somatic mosaicism. The mutagenic potential of ASD toxicants may be further potentiated by intrinsic genomic instability among ASD probands, with recent identification of rare tandem repeat expansions among affected individuals likely increasing mutational liability to environmental toxins. Whereas iPSC derived neuronal lines might now be used to test toxin exposure on mutation rates [116], determining the specific contribution of these environmentally-induced DNA alterations to ASD is difficult, as there is a paucity of population-based, longitudinal evidence necessary to draw conclusive links between exposure, genotypic responses, and phenotypic consequences. In addition, neglect for the critical influence of paternal exposures on offspring outcomes is evident in available epidemiological surveys of ASD trends, creating a maternally-biased view of the contribution of environment to the disorder phenotype. The development of comprehensive prospective birth cohorts in tandem with increasing fidelity and accessibility of high-throughput sequencing offers unprecedented opportunities to trace the effects and outcomes of developmental genotoxin exposure. This will deepen our understanding of the complex gene-environment relationships underpinning the aetiology of ASD.

## Supplementary information

Supplementary Information
