# Supplementary material for: Environmental exposures associated with elevated risk for autism spectrum disorder may augment the burden of deleterious de novo mutations among probands
Source: Mol Psychiatry. 2021 May 17;27(1):710–30. doi: 10.1038/s41380-021-01142-w (PMC8960415; doi:10.1038/s41380-021-01142-w)
Supplement: Supplementary file 1 — Supplementary Information [file 41380_2021_1142_MOESM1_ESM.docx]

**Supplementary Information**

**Search Terms & Search Strategy**

Evidence listed in Table 2 was compiled via a comprehensive search of PubMed, SCOPUS, and Google Scholar databases for relevant, full-length articles published after 2000, with prioritisation of those made available after 2010, in which the most recent literature review was performed (Kinney et al., 2010), and up to the date of the 1^st^ June 2020. Inclusion of papers published between 2000 to 2010 was deemed necessary where epidemiological follow-up studies of exposure risk had not been performed in the subsequent decade, or where critical evidence of genotoxicity/mutagenicity of exposures not previously reviewed by Kinney et al. (2010) was unavailable. A primary list of agents associated with ASD was provided by a recent publication observing the selective targeting of ASD-associated genes by environmental pollutants (Carter & Blizard, 2016). To confirm their association with ASD, each agent was searched in combination with the following terms: (“autism” OR “Asperger” OR “ASD” OR “autism spectrum disorder” OR “pervasive developmental delay” OR “PDD”). The bibliographies of articles identified in the searches, including reviews and peer reviewed research, were also searched for additional environmental agents not included in the primary list. Exposures associated with ASD beyond the perinatal period were deemed beyond the scope of the review and excluded. The final list of environmental agents of interest consisted of the following: (1,2,4-trichlorobenzene), (1,3-butadiene), (1,4-dioxane), (2,3,7,8-Tetrachlorodibenzo-p-dioxin), (Acetaldehyde), (Age), (Aluminium), (Antibiotics), (“Antidepressant medication”), (Antimony), (Arsenic), (Benzene), (“Benzo(a)pyrene”), (Beryllium), (“Bisphenol A”), (Bromoform), (Cadmium), (Cannabis), (“Carbon Monoxide”), (Chlordan), (Chromium), (Cocaine), (Copper), (dexamethasone), (Dibenzofurans), (“Dichlorodiphenyldichloroethylene” OR “DDE”), (“Dichlorodiphenyltrichloroethane” OR “DDT”), (Diesel*), (Ethanol), (“Ethylene dichloride”), (“Folate” OR “B9”), (Formaldehyde), (“Glycol Ether”), (Glyphosate), (Iron), (Lead), (Manganese), (“Maternal Diabetes” OR “Maternal Obesity”), (Mercury), (“Methyl Tert Butyl Ether” OR “MTBE”), (“Methylene Chloride” OR “Dichloromethane”), (Molybdenum), (Nickel), (“Nicotine” OR “Smoking”), (Opioid), (Organochlorine), (Organophosphate), (Oxytocin), (“Paracetamol” OR “Acetaminophen”), (“Particulate matter”), (Perchlorate), (Phthalates), (“Polybrominated Diphenyl”), (“Polychlorinated Biphenyl”), (Propionaldehyde), (Prostaglandins), (Pyrethroids), (Quinoline), (Styrene), (Terbutaline), (“Tetrachloroethylene” OR “Perchloroethylene”), (Thalidomide), (Tin), (Toluene), (Trichloroethylene), (Tungsten), (Valproate), (Vanadium), (“Vinyl Chloride”), (Vitamin), (Xylene), (Zinc). Evidence of mutagenicity/genotoxicity for the identified agents was determined by combining each of the above with the following search terms: (“mutagen*” OR “oxidative stress” OR “oxidative species” OR “ROS” OR “genotoxic*” OR “clastogenic*” OR “aneugenic” OR “DNA repair”). Only environmental agents demonstrating evidence of mutagenicity/genotoxicity in human or other mammalian models were included in the final review. Studies replicating the mutagenic/genotoxic effect in identical *in vivo/ in vitro* were excluded to avoid extensive repetition.
